# miR‐145 inhibits the proliferation and migration of vascular smooth muscle cells by regulating autophagy

**DOI:** 10.1111/jcmm.15316

**Published:** 2020-04-26

**Authors:** Weirong Wang, Lifang Chen, Chenxu Shang, Zhen Jin, Feng Yao, Liang Bai, Rong Wang, Sihai Zhao, Enqi Liu

**Affiliations:** ^1^ Department of Medical Laboratory Animal Science School of Basic Medical Sciences Xi’an Jiaotong University Health Science Center Xi’an China; ^2^ Research Institute of Atherosclerotic Disease Xi’an Jiaotong University Cardiovascular Research Center Xi’an China; ^3^ Department of Pharmacology School of Basic Medical Sciences Xi’an Jiaotong University Health Science Center Xi’an China

**Keywords:** autophagy, migration, miR‐145, proliferation, VSMCs

## Abstract

miR‐145, the most abundant miRNA in the vascular smooth muscle cells (VSMCs), regulates VSMC function in intimal hyperplasia. It has been reported that autophagy participates in the regulation of proliferation and migration of VSMCs. However, the effect of miR‐145 on autophagy and related mechanism in the proliferation and migration of VSMCs remains unclear. Therefore, we aimed to determine the effect of miR‐145 on autophagy and the mechanism in VSMCs. Cell autophagy was determined by transmission electron microscope, mRFP‐GFP‐LC3 assay and Western blotting. A recombinant lentivirus containing miR‐145 was used to construct VSMCs with miR‐145 overexpression. We found that miR‐145 expression was decreased, and autophagy was increased in the carotid arteries of C57BL/6J mice with intimal hyperplasia and TGF‐β1‐stimulated VSMCs. Furthermore, miR‐145 overexpression inhibited cell autophagy, whereas miR‐145 inhibition promoted autophagy in TGF‐β1‐stimulated VSMCs. Meanwhile, miR‐145 inhibited the proliferation and migration of VSMCs. More importantly, our study showed that autophagy inhibition augmented the inhibitory effect of miR‐145 on the proliferation and migration of VSMCs. In addition, we found that the sirtuins are not direct targets of miR‐145 in the proliferation and migration of VSMCs. These results suggest that miR‐145 inhibits the proliferation and migration of VSMCs by suppressing the activation of autophagy.

## 1 INTRODUCTION

microRNA‐145 (miR‐145) is a 22‐nt, highly conserved miRNA. It is generally accepted that miR‐145 has a strong inhibitory effect on the proliferation and migration of cancer cells and is a tumour suppressor.^1^, ^2^ Cheng *et al* found that miR‐145 is the most abundant miRNA in vascular walls, and miR‐145 is selectively expressed in the vascular smooth muscle cells (VSMCs) of vascular walls.^3^ Subsequent studies demonstrated that miR‐145 participates in the regulation of VSMC function including the proliferation and migration in intimal hyperplasia.^4^, ^5^

Autophagy is an important biological process and plays a crucial role in cellular homeostasis in cardiovascular diseases.^6^ Autophagy is generally recognized as an important mediator of VSMC function.^7^ Several studies have indicated that the activation of autophagy contributes to the proliferation and migration of VSMCs. Li *et al* showed that sonic hedgehog induced cell autophagy and resulted in an increase in VSMC proliferation, which plays a key role in the pathogenesis of neointima formation.^8^ Another study showed that platelet‐derived growth factor (PDGF) induced autophagy and that inhibition of autophagy by 3‐methyladenine (3‐MA) reduced PDGF‐induced proliferation and migration of VSMCs.^9^ However, the effect of miR‐145 on autophagy and the underlying mechanism in the proliferation and migration of VSMCs remains unclear.

miR‐145 exerts biological functions, including the modulation of VSMC proliferation and migration, via its multiple target genes. It has been reported that Krüppel‐like factor 5 (KLF5), TGFβ receptor II (TGFBR2) and CD40 were the direct targets of miR‐145 in the proliferation and phenotypic modulation of VSMCs.^3^, ^10^, ^11^ Sirtuins are a family of evolutionally conserved class III histone deacetylases. The mammalian sirtuin family includes seven members (SIRT1‐7).^12^ Emerging evidence indicates that sirtuins are also the targets of miRNAs in cardiovascular diseases. A recent report showed that SIRT1 was the target of miR‐34a in the differentiation of SMCs from pluripotent stem cells.^13^ Zhu* et al* reported that miR‐195 augmented palmitate‐induced apoptosis of cardiomyocytes by targeting SIRT1.^14^ In addition, miR‐497 inhibited cardiac hypertrophy by targeting SIRT4.^15^ Therefore, we speculate that miR‐145 is likely able to regulate the proliferation and migration of VSMCs by targeting sirtuins.

In this study, we first determined the change of miR‐145 and autophagy in mice with intimal hyperplasia and VSMCs stimulated with TGF‐β1. Then, we investigated the effect of miR‐145 on autophagy and the related mechanism in the proliferation and migration of VSMCs.

## 2 MATERIALS AND METHODS

### 2.1 Materials

TGF‐β1 was purchased from PeproTech (Rocky Hill, NJ, USA). The cell counting kit‐8 (CCK‐8) was obtained from Dojindo Molecular Technologies (Dojindo Laboratories, Kumamoto, Japan). 3‐MA was ordered from Selleckchem (Houston, TX, USA). Antibodies against LC3, p62, SIRT1, SIRT3, SIRT5 and β‐actin were purchased from Cell Signaling Technology (Beverly, MA, USA). Antibodies against Beclin1, proliferating cell nuclear antigen (PCNA) and SIRT6 were obtained from Abcam (Cambridge, MA, USA). The mir‐X™ miRNA First‐Strand Synthesis and SYBR qRT‐PCR kits were purchased from Clontech Laboratories, Inc (Mountain View, CA, USA). RNAiso Plus, PrimeScript RT Master Mix and SYBR Premix Ex Taq II were ordered from Takara Bio Company (Takara, Shiga, Japan). The tandem fluorescent‐tagged LC3 (mRFP‐GFP‐LC3) was obtained from Hanheng Biotechnology, Inc (Shanghai, China). Lipofectamine 2000 transfection reagent was purchased from Invitrogen (Carlsbad, CA, USA). The X‐tremegene HP DNA transfection reagent was purchased from Roche Diagnostics (Indianapolis, IN, USA). The luciferase reporter assay system was purchased from Promega (Madison, WI, USA). All other chemicals were ordered from commercial sources.

### 2.2 Animals and treatment

Male C57BL/6J mice, weighing 22‐24 g, were provided by the Laboratory Animal Center of Xi'an Jiaotong University. Mice were anesthetized, and the carotid arteries were dissected after a midline cervical incision. For the ligation model, the carotid arteries just proximal to the bifurcation were ligated with silk sutures.^16^ Right carotid arteries were used as the control. After 4 weeks, both carotid arteries were harvested. For Western blotting and real‐time PCR analysis, the carotid arteries were snap‐frozen in liquid nitrogen. For haematoxylin and eosin (H&E) staining, the carotid arteries were processed into 7‐μm thick serial frozen sections. The wall thickness of carotid arteries was measured using the Image‐Pro Plus (IPP) software (Media Cybernetics, Bethesda, MD, USA). The experimental protocol was in accordance with the National Institutes of Health Guide for Care and Use of Laboratory Animals and was approved by the Laboratory Animal Care Committee of Xi'an Jiaotong University.

### 2.3 VSMC culture and characterization

Male Sprague Dawley rats ranging from 150 to 180 g were from Laboratory Animal Center of Xi'an Jiaotong University. The primary VSMCs were isolated from the thoracic aorta of rats by the tissue explant method.^17^ Briefly, the thoracic aorta was removed and longitudinally opened, and it was then washed with cold phosphate‐buffered saline (PBS) under sterile conditions. The adventitia and intima were separated from the media, and the isolated media was cut into pieces and placed in DMEM supplemented with 20% foetal calf serum. The cells were characterized by immunofluorescence staining with α‐actin antibody. Cells at passages 3‐9 were used for experiments.

### 2.4 Cell proliferation assay

VSMC proliferation was evaluated by cell counting and the CCK‐8 assays. Cell counting was performed by the trypan blue exclusion method.^18^ For the CCK‐8 assay, VSMCs was added 10 μL of CCK‐8 to each well of 96‐well plates for 2 h, and the absorbance at 450 nm was measured using a microplate reader (Thermo Fisher, Waltham, MA, USA).^19^

### 2.5 Cell migration assay

Cell migration was assessed by the wound‐healing assay.^20^ VSMCs were cultured in 6‐well plates at 90% confluence. The monolayer was scratched with a pipette tip. Images at time zero (*t* = 0 h) were photographed by a microscope (Nikon, Tokyo, Japan) to record the initial area of the wounds. The cells were then cultured for 24 h, and the widths of the wounds were also captured (t = 24 h). The wound‐healing width was determined by the IPP software (Media Cybernetics, Bethesda, MD, USA). Wound diameter was measured at two time points (0 h and 24 h) to assess % wound healing at 24 h using the formula:

(Width at 0 h ‐ Width at 24 h)/ (Width at 0 h) X 100%

### 2.6 Western blotting

The total protein of VSMCs was extracted with RIPA lysis buffer containing protease inhibitor cocktail. Protein samples were separated by SDS‐PAGE and transferred to PVDF membranes. After blocking with 5% nonfat dry milk, the membranes were incubated with primary antibodies overnight. The primary antibodies used were anti‐PCNA (1:400), anti‐LC3 (1:1000), anti‐Beclin1 (1:1000), anti‐p62 (1:1000), anti‐SIRT1 (1:1000), anti‐SIRT3 (1:1000), anti‐SIRT5 (1:1000) anti‐SIRT6 (1:1000) and anti‐β‐actin (1:1000). The membranes were then incubated for 2 h at room temperature with HRP‐conjugated secondary IgG (1:5000). The relative intensity of protein bands was quantified by the Quantity One Analysis Software (BioRad, Hercules, CA, USA), and β‐actin was used as an internal control.

### 2.7 Quantitative real‐time PCR

Total RNA was extracted from cells using the RNAiso Plus reagent. cDNA was synthesized using the PrimeScript RT Master Mix. cDNA was amplified using the SYBR Premix Ex Taq II. GAPDH was used as an endogenous control. Data were normalized to the GAPDH mRNA level.

miR‐145 expression was determined by the mir‐X™ miRNA First‐Strand Synthesis and SYBR qRT‐PCR kits according to the manufacturer's instructions. U6 was used as an endogenous control, and relative gene expression was quantitatively analysed by the comparative Ct method (2^−△△CT^). Data were normalized to the U6 mRNA level.

### 2.8 mRFP‐GFP‐LC3 assay

The cells were infected with adenovirus harboring mRFP‐GFP‐LC3. Twelve hours after adenovirus infection, the cells were treated with TGF‐β1 for 24 h. The results were visualized using super‐resolution confocal microscope (Leica, Mannheim, BW, Germany).

### 2.9 Transmission electron microscope

The cells were collected by trypsinization and centrifugation and then were fixed with 2.5% glutaraldehyde and 1% osmium tetroxide followed by dehydration in an increasing series of ethanol. The samples were embedded in Durcopan ACM for 6 h, and ultrathin sections were cut using a Leica Ultramicrotome EM UC6, The sections were then stained with uranyl acetate and lead citrate, and examined with a Tecnai G^2^ 12 transmission electron microscope (FEI Company, Holland).

### 2.10 Generation of stable cell lines expressing miR‐145

The recombinant lentivirus of miR‐145 (LV‐miR‐145) and the negative control lentivirus (LV‐NC) were constructed by Genechem Co., Ltd. (Shanghai, China). VSMCs were seeded in 24‐well plates for 24 h. Then, the lentivirus was added to the cells with polybrene and an enhanced infection solution for 24 h, followed by incubation in fresh DMEM media for the next 48 h. The infection efficiency was detected by a fluorescence microscopy analysis of GFP expression.

### 2.11 miR‐145 inhibition

The miR‐145 inhibitor was obtained from GenePharma Co., Ltd. (Shanghai, China). The miR‐145 inhibitor and Lipofectamine 2000 were separately diluted in serum‐free DMEM and incubated at room temperature. The two solutions were gently mixed and incubated for 20 min and then were added to the cells. VSMCs transfected with the miR‐145 inhibitor were incubated for an additional 48 h at 37°C in a CO_2_ incubator prior to experimental use.

### 2.12 Ingenuity pathway analysis

Ingenuity pathway analysis was performed by Shanghai Cloud Scientific Technology Co., Ltd. (Shanghai, China).

### 2.13 Luciferase reporter assay

A luciferase reporter assay was performed to confirm the target genes of miR‐145. Briefly, HEK‐293T cells were transfected at 60% confluency in 24‐well plates with wild‐type or mutant 3’‐UTR vectors and miR‐145 or miR‐145 control vectors. A co‐transfected Renilla luciferase reporter vector was used as an internal control for the normalization of luciferase activity in each sample. Cells were analysed at 48 h after transfection. Firefly and Renilla luciferase activities were quantified in lysates using the luciferase reporter assay kit. The firefly luciferase enzyme activity was normalized to the Renilla luciferase enzyme activity.

### 2.14 Statistical analysis

Data are presented as the mean ± SEM from three independent experiments. Statistical significance was determined using one‐way analysis of variance. *P* < 0.05 was considered statistically significant.

## 3 RESULTS

### 3.1 The changes of miR‐145 and autophagy in mice with intimal hyperplasia

**Figure 1:**
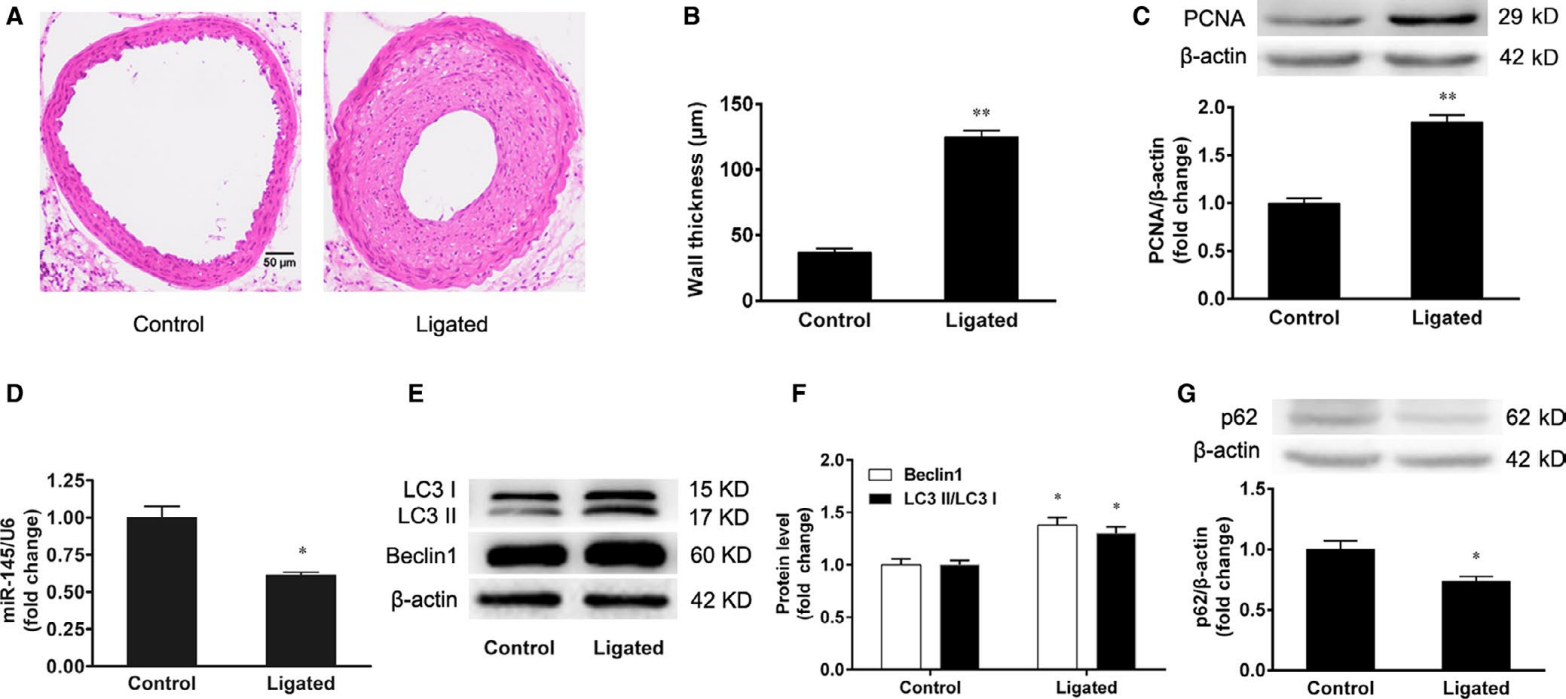
The changes of miR‐145 and autophagy in mice with intimal hyperplasia. The left carotid arteries of male C57BL/6J mice were ligated with silk suture. The right carotid arteries were used as the control. After 28 days, both carotid arteries were harvested. A, B, Representative sections of control and ligated arteries stained with H&E and the quantification data of carotid artery thickness (Scale bar = 50 μm), n = 8. C, The expression of PCNA protein in carotid arteries was detected by Western blotting. D, The expression of miR‐145 in carotid arteries was detected by real‐time PCR. E, F, The conversion of LC3 I to LC3 II and the expression of Beclin1 in carotid arteries were detected by Western blotting. G, The expression of p62 in carotid arteries was detected by Western blotting. The results are expressed as the mean ± SEM, n = 3. Statistical significance was determined using ANOVA by Student's *t* test. *P < 0.05 and **P < 0.01 vs. the control group

The left carotid arteries were ligated, and the right carotid arteries were used as the control in C57BL/6J mice. To observe the intimal hyperplasia, the carotid arteries were stained with H&E at 28 days after ligation. Our results showed that there was a significant intimal thickening in the ligated carotid arteries (Figure 1A,B). Moreover, the protein expression of PCNA in the ligated carotid arteries was increased compared with the control carotid arteries (*P* < 0.01) (Figure 1C). More importantly, miR‐145 expression was decreased in the ligated carotid arteries (Figure 1D). Our results also showed that the conversion of LC3 I to LC3 II and Beclin1 protein expression were increased in the ligated carotid arteries compared with that of the control carotid arteries (Figure 1E,F). In addition, we found that the p62 protein expression was decreased in the ligated carotid arteries (Figure 1G). These results suggest that miR‐145 expression is decreased and autophagy is increased in the carotid arteries of mice with intimal hyperplasia.

### 3.2 The effect of TGF‐β1 on autophagy in VSMCs

**Figure 2:**
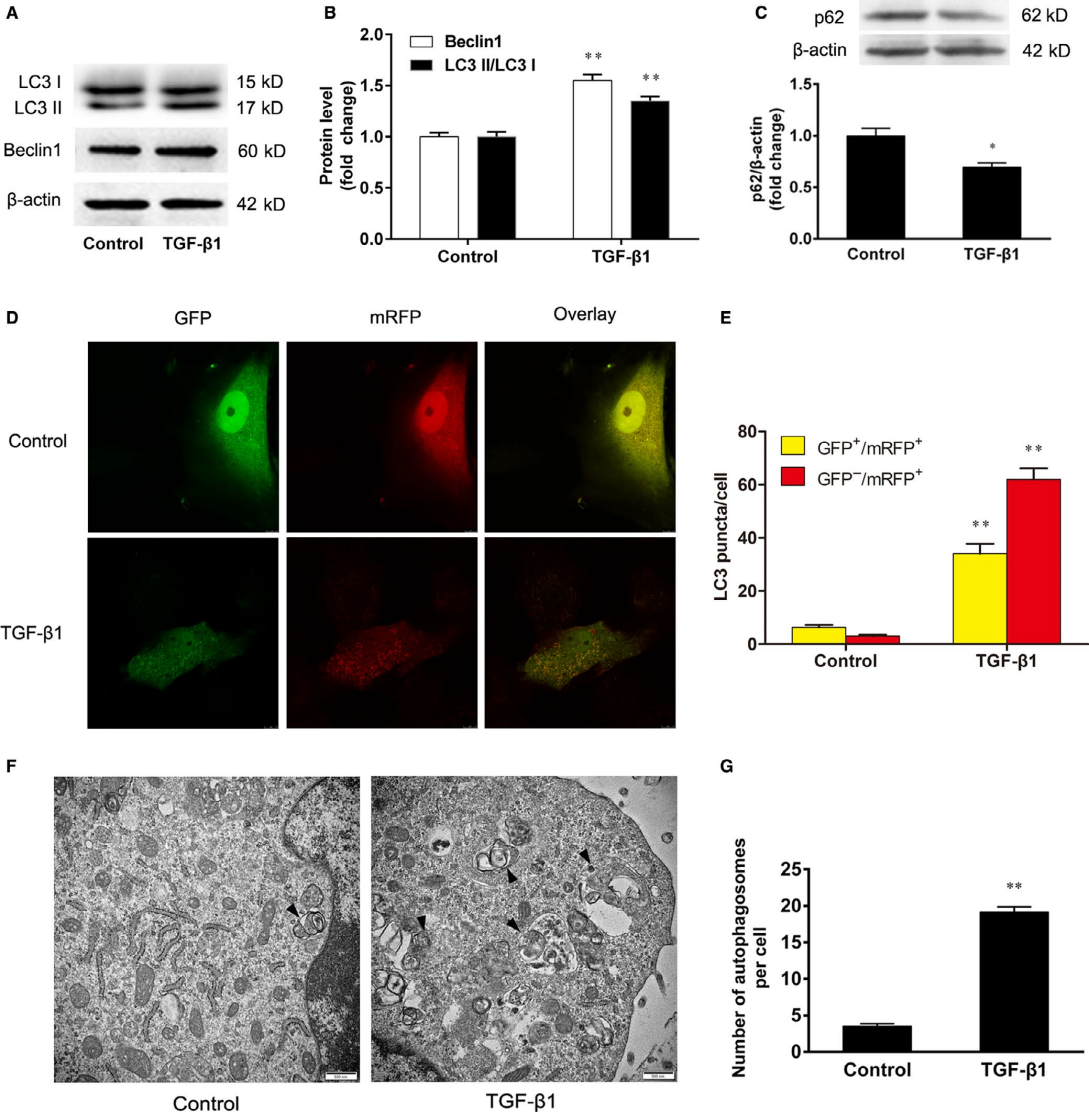
The effect of TGF‐β1 on autophagy in VSMCs. The VSMCs were treated with TGF‐β1 (5 ng/mL) for 24 h. A, B, The conversion of LC3 I to LC3 II and the expression of Beclin1 were determined by Western blotting. C, The expression of p62 was determined by Western blotting. D, E, The VSMCs were infected with mRFP‐GFP‐LC3 adenovirus for 12 h, and then the cells were treated with TGF‐β1 for 24 h. The merged images (yellow) show overlay of GFP‐LC3 (green) and mRFP‐LC3 (red). The accumulation of red and yellow puncta were quantified for both control and TGF‐β‐treated cells. F, G, The autophagosome formation was analysed by transmission electron microscopy. Solid black arrowheads indicate the presence of autophagosomes. At least 20 cells were collected for statistical analysis in each group. The results are expressed as the mean ± SEM, n = 3. Statistical significance was determined using ANOVA by Student's t test. *P < 0.05 and **P < 0.01 vs. the control group

To investigate the effect of TGF‐β1 on autophagy, VSMCs were treated with TGF‐β1 (5 ng/mL) for 24 h, and the conversion of LC3 I to LC3 II and Beclin1 expression were determined by Western blotting. We found that TGF‐β1 increased the conversion of LC3 I to LC3 II and Beclin1 protein expression in VSMCs (Figure 2A,B). Meanwhile, TGF‐β1 decreased p62 protein expression of VSMCs (Figure 2C). Then, we performed the mRFP‐GFP‐LC3 assay to observe the autophagic flux in VSMCs. When autophagy was induced by TGF‐β1, the red puncta were accumulated in VSMCs compared with the control group (*P* < 0.01) (Figure 2D,E). The result showed that TGF‐β1 activated the autophagic flux in VSMCs. To further confirm these results, the autophagosome formation was assessed by transmission electron microscopy. We found that TGF‐β1 increased the autophagosome formation in VSMCs compared with the control group (Figure 2F,G). These data demonstrate that TGF‐β1 induces VSMC autophagy by increasing the autophagic flux and the conversion and expression of LC3 and Beclin1, and autophagosome formation.

### 3.3 miR‐145 regulates autophagy of VSMCs

**Figure 3:**
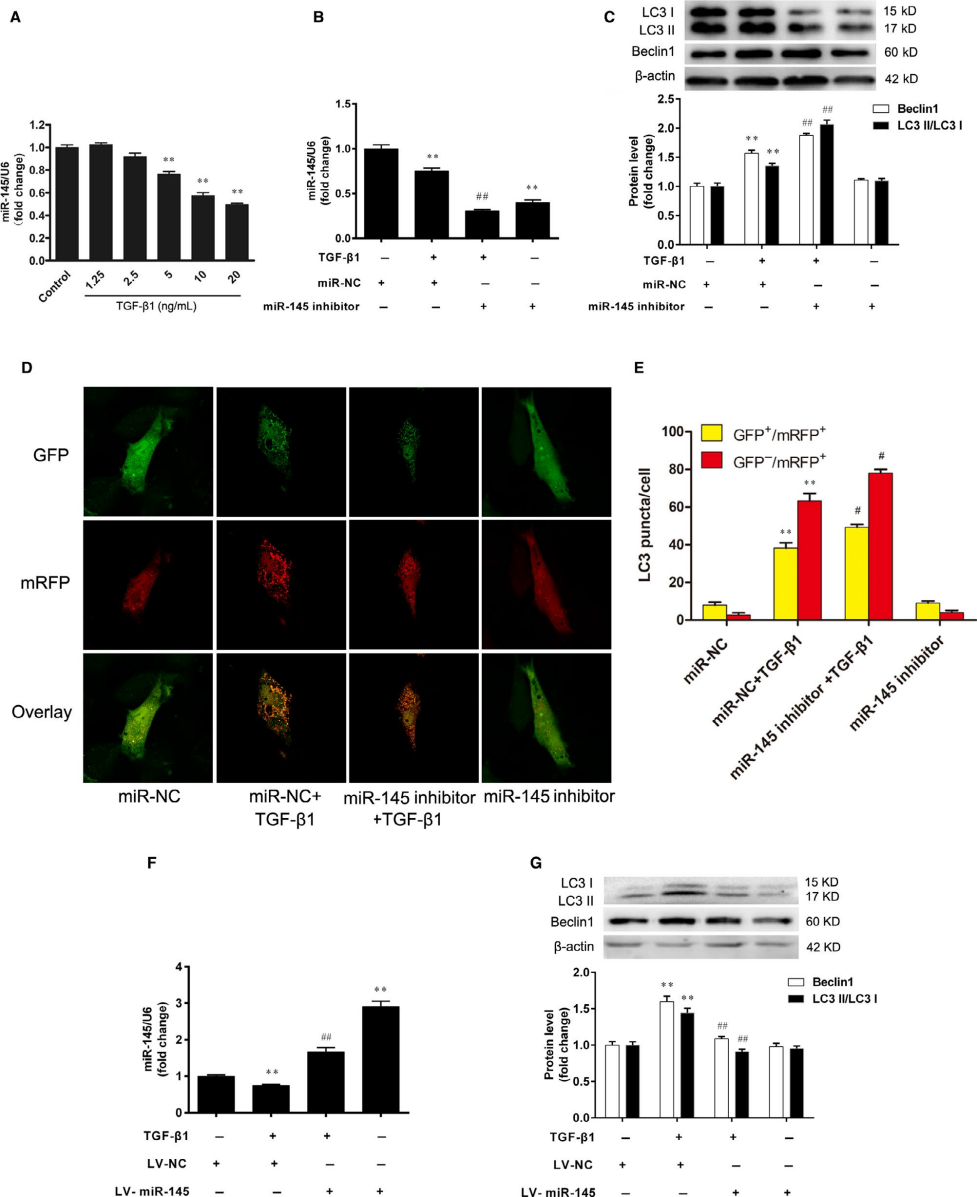
miR‐145 regulates autophagy of VSMCs. A, The VSMCs were treated with TGF‐β1 (1.25, 2.5, 5, 10 and 20 ng/mL) for 24 h, and the expression of miR‐145 was assessed by real‐time PCR. **P < 0.01 vs. the control group. The miR‐145 inhibitor or negative control (miR‐NC) were transfected into VSMCs for 48 h, prior to treatment with TGF‐β1 (5 ng/mL) for an additional 24 h. B, The expression of miR‐145 was assessed by real‐time PCR. C–E, The autophagy was determined by Western blotting and mRFP‐GFP‐LC3 assay. **P < 0.01 vs. the miR‐NC group. ^#^ *P* < 0.05 and ^##^ *P* < 0.01 vs. the miR‐NC + TGF‐β1 group. The VSMCs infected with recombinant lentivirus of miR‐145 (LV‐miR‐145) or the negative control lentivirus (LV‐NC) were treated with TGF‐β1 (5 ng/mL) for 24 h. F, The expression of miR‐145 was assessed by real‐time PCR. (G) The autophagy was determined by Western blotting. **P < 0.01 vs. the LV‐NC group. ^##^ *P* < 0.01 *vs.* the LV‐NC + TGF‐β1 group. The results are expressed as the mean ± SEM, n = 3. Statistical significance was determined using ANOVA by Tukey's post hoc test

The VSMCs were treated with TGF‐β1 (1.25, 2.5, 5, 10 and 20 ng/mL) for 24 h, and the expression of miR‐145 was assessed by real‐time PCR. We found that TGF‐β1 decreased miR‐145 expression in VSMCs. There was a significant difference between the TGF‐β1 (5, 10 and 20 ng/mL) groups and the control group (*P* < 0.01) (Figure 3A).

Given our findings that TGF‐β1 promoted cell autophagy and decreased miR‐145 expression in VSMCs, we explored the possibility whether miR‐145 could regulate autophagy. The results demonstrated that the miR‐145 inhibitor significantly reduced miR‐145 expression (Figure 3B). Furthermore, miR‐145 inhibitor enhanced the conversion of LC3 I to LC3 II and up‐regulated Beclin1 expression in TGF‐β1‐stimulated VSMCs (Figure 3C). The mRFP‐GFP‐LC3 assay also showed that miR‐145 inhibitor activated the autophagic flux in the TGF‐β1‐stimulated VSMCs (Figure 3D and E). To further confirm the effect of miR‐145 on VSMC autophagy, VSMCs were infected with LV‐miR‐145 or LV‐NC and then were treated with TGF‐β1 (5 ng/mL) for 24 h. The results showed that miR‐145 expression was approximately increased by threefold in VSMCs infected with LV‐miR‐145 (Figure 3F). By contrast, overexpression of miR‐145 in VSMCs decreased the conversion of LC3 I to LC3 II and Beclin1 expression compared with the TGF‐β1 group (Figure 3G). These results suggest that miR‐145 attenuated the activation of autophagy in VSMCs stimulated with TGF‐β1.

### 3.4 TGF‐β1 promotes the proliferation and migration of VSMCs

**Figure 4:**
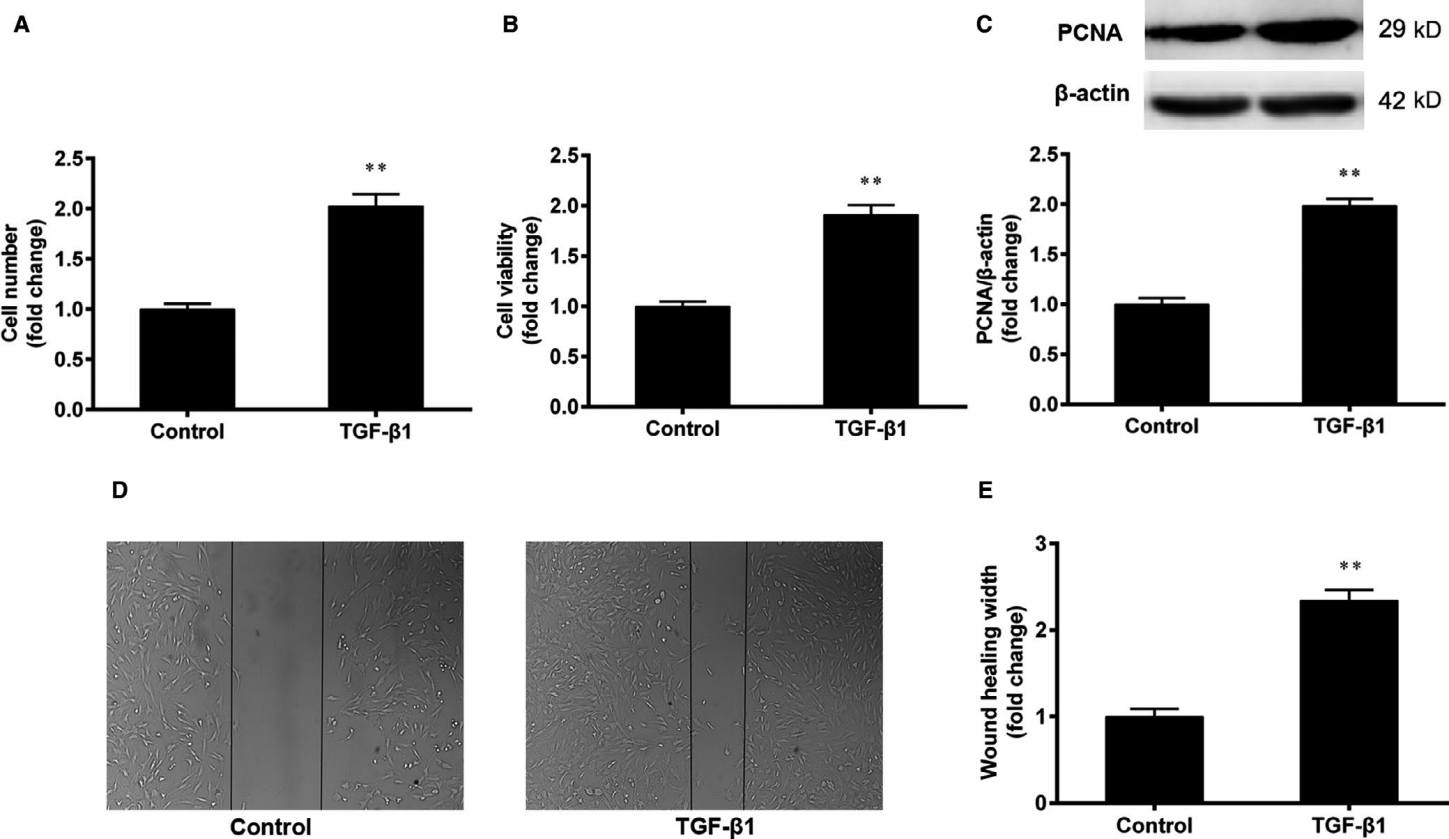
TGF‐β1 promotes the proliferation and migration of VSMCs. The VSMCs were treated with TGF‐β1 (5 ng/mL) for 24 h. A, B, Cell proliferation was evaluated by cell counting and the CCK‐8 assays. C, The expression of PCNA protein was determined by Western blotting. D, E, The migration of VSMCs was determined by the wound‐healing assay. The results are expressed as the mean ± SEM, n = 3. Statistical significance was determined using ANOVA by Student's *t* test. **P < 0.01 vs. the control group

To confirm the effect of TGF‐β1 on VSMC proliferation, the VSMCs were treated with TGF‐β1 (5 ng/mL) for 24 h. Cell proliferation was evaluated by cell counting and the CCK‐8 assays. The expression of PCNA protein was determined by Western blotting. As shown in Figure 4A,B, TGF‐β1 increased both the cell number and cell viability compared with the control group (*P* < 0.01). In addition, the expression of PCNA protein was also increased (Figure 4C). These results collectively show that TGF‐β1 promotes the proliferation of VSMCs.

The effect of TGF‐β1 on the migration of VSMCs was determined by the wound‐healing assay. As shown in Figure 4D,E, TGF‐β1 decreased the width of the scratched wound and increased the wound‐healing width, compared with the control group (*P* < 0.01). The result show that TGF‐β1 promotes the migration of VSMCs.

### 3.5 miR‐145 inhibits the proliferation and migration of VSMCs

**Figure 5:**
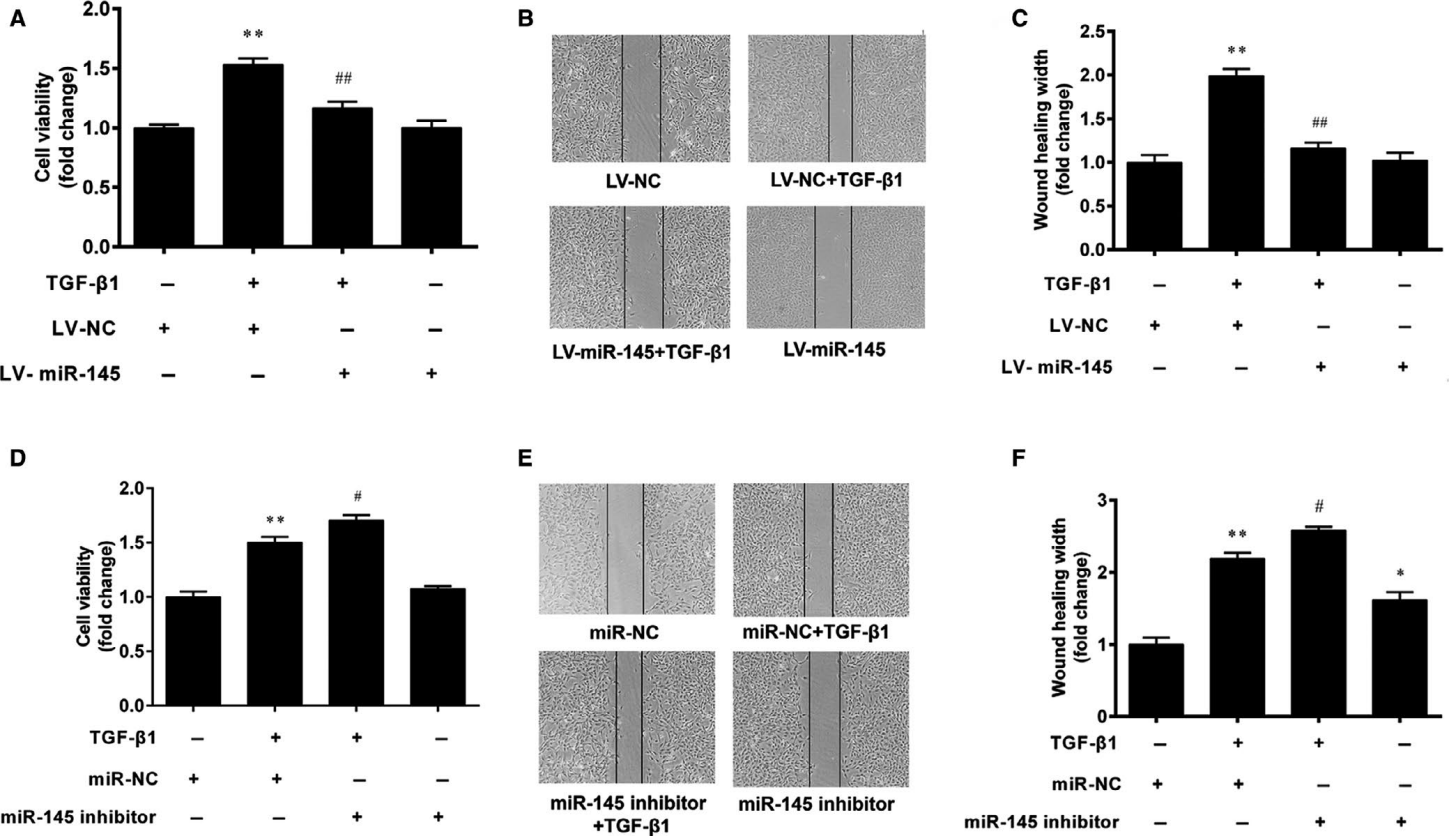
miR‐145 inhibits the proliferation and migration of VSMCs. The VSMCs infected with LV‐miR‐145 or LV‐NC were treated with TGF‐β1 (5 ng/mL) for 24 h. A, Cell proliferation was evaluated by the CCK‐8 assay. B, C, Cell migration was determined by the wound‐healing assay. **P < 0.01 vs. the LV‐NC group. ^##^ *P* < 0.01 vs. the LV‐NC + TGF‐β1 group. The miR‐145 inhibitor or miR‐NC were transfected into VSMCs for 48 h, prior to treatment with TGF‐β1 for an additional 24 h. D, Cell viability was determined by the CCK‐8 assay. E, F, Cell migration was determined by the wound‐healing assay. *P < 0.05 and **P < 0.01 vs. the miR‐NC group. ^#^ *P* < 0.05 vs. the miR‐NC + TGF‐β1 group. The results are expressed as the mean ± SEM, n = 3. Statistical significance was determined using ANOVA by Tukey's post hoc test

The above results showed that TGF‐β1 decreased miR‐145 expression and promoted the proliferation and migration of VSMCs. Next, we investigated whether miR‐145 could regulate VSMC proliferation and migration. VSMCs infected with LV‐miR‐145 or LV‐NC were then treated with TGF‐β1 (5 ng/mL) for 24 h. The result of CCK‐8 assay showed that miR‐145 overexpression decreased the cell viability of VSMCs stimulated with TGF‐β1 (Figure 5A). Meanwhile, miR‐145 overexpression inhibited the migration of VSMCs (Figure 5B,C).

To further verify the effect of miR‐145 on VSMC proliferation and migration, the miR‐145 inhibitor was transfected into VSMCs. The result showed that the miR‐145 inhibitor promoted TGF‐β1‐induced proliferation of VSMCs (Figure 5D). Furthermore, the result of the wound‐healing assay also showed that the miR‐145 inhibitor further increased the wound‐healing width in VSMCs stimulated with TGF‐β1 (*P* < 0.05) (Figure 5E,F).

### 3.6 miR‐145 inhibits the proliferation and migration of VSMCs through autophagy

**Figure 6:**
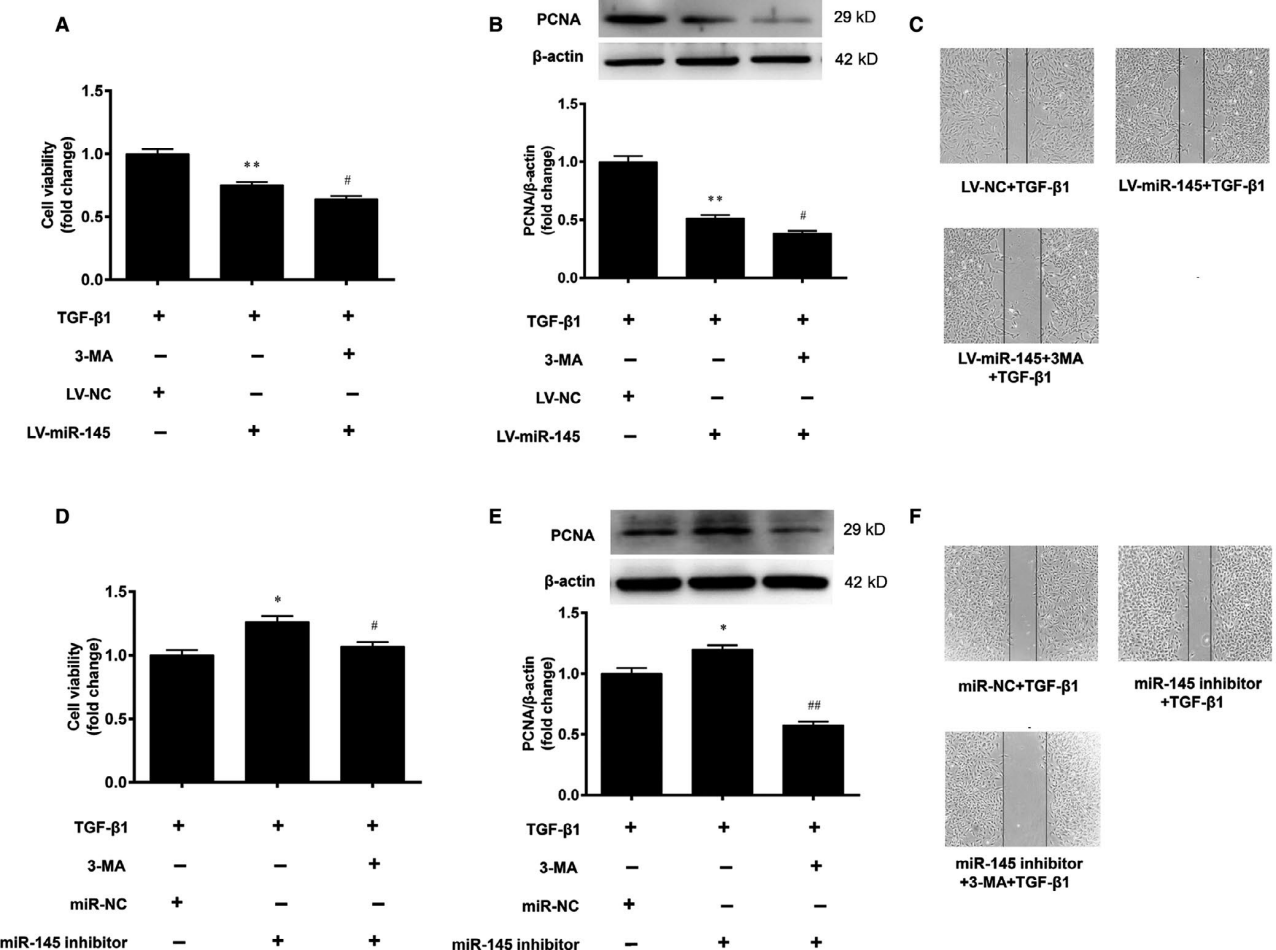
miR‐145 regulates the proliferation and migration of VSMCs through autophagy. A–C, The VSMCs with miR‐145 overexpression were pretreated with the autophagy inhibitor 3‐MA for 1 h before stimulating with TGF‐β1 (5 ng/mL), and the cell viability, PCNA protein expression and cell migration were determined. **P < 0.01 vs. the LV‐NC + TGF‐β1 group. ^#^ *P* < 0.05 vs. the LV‐miR‐145 + TGF‐β1 group. D‐F, The VSMCs were transfected with the miR‐145 inhibitor for 48 h, followed by application of 3‐MA for 1 h before stimulating with TGF‐β1 and the cell viability, PCNA protein expression and cell migration were determined. *P < 0.05 vs. the miR‐NC + TGF‐β1 group. ^#^ *P* < 0.05 and ^##^ *P* < 0.01 vs. the miR‐145 inhibitor + TGF‐β1 group. The results are expressed as the mean ± SEM, n = 3. Statistical significance was determined using ANOVA by Tukey's post hoc test

To evaluate whether miR‐145 regulates the proliferation and migration of VSMCs through autophagy, the VSMCs were pretreated with the autophagy inhibitor 3‐MA (5 mM) for 1 h prior to TGF‐β1. Compared with the miR‐145 overexpression group, 3‐MA further decreased cell viability and the expression level of PCNA (Figure 6A,B). Moreover, 3‐MA decreased the wound‐healing width in VSMCs stimulated with TGF‐β1 (Figure 6C). These results suggest that autophagy inhibition augmented the inhibitory effects of miR‐145 on the proliferation and migration of VSMCs. In addition, we also found that the promotive effects of miR‐145 inhibitor on the proliferation and migration of VSMCs were significantly attenuated by 3‐MA (Figure 6D‐F). These data demonstrate that miR‐145 regulates the proliferation and migration of VSMCs through autophagy.

### 3.7 Sirtuins are not the direct targets of miR‐145

**Figure 7:**
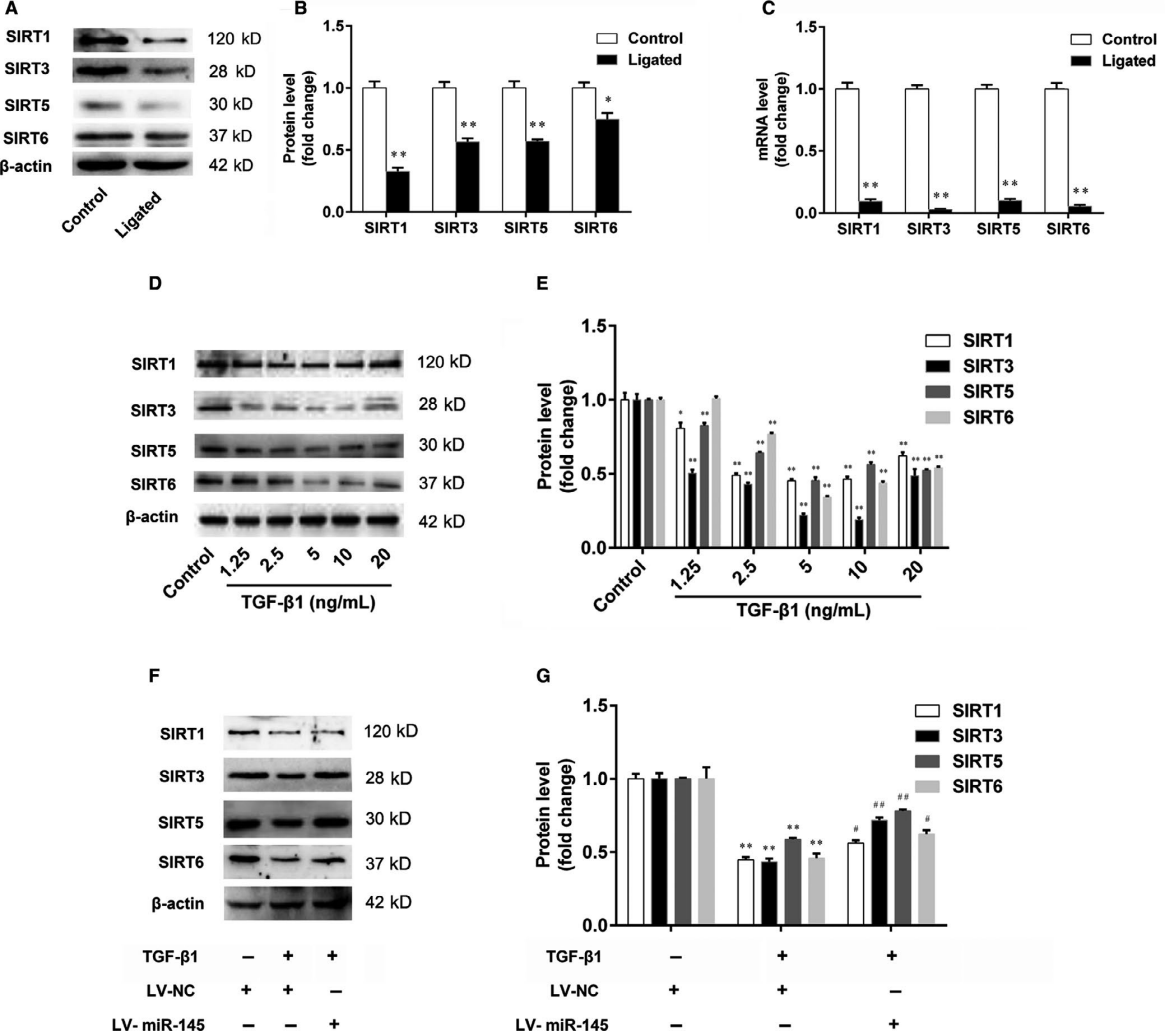
The expression of sirtuins in the carotid arteries and VSMCs. A‐C, The expression levels of SIRT1, SIRT3, SIRT5 and SIRT6 in carotid arteries were determined by Western blotting and real‐time PCR, respectively. D, E, The VSMCs were treated with TGF‐β1 (1.25, 2.5, 5, 10 and 20 ng/mL) for 24 h, and the expression levels of SIRT1, SIRT3, SIRT5 and SIRT6 in VSMCs were determined by Western blotting. Statistical significance was determined using ANOVA by Student's *t* test. *P < 0.05 and **P < 0.01 vs. the control group. (F and G) The effect of miR‐145 on the expression of SIRT1, SIRT3, SIRT5 and SIRT6 in VSMCs was determined by Western blotting. Statistical significance was determined using ANOVA by Tukey's post hoc test. **P < 0.01 vs. the LV‐NC group. ^#^ *P* < 0.05 and ^##^ *P* < 0.01 vs. the LV‐NC + TGF‐β1 group. The results are expressed as the mean ± SEM, n = 3

To investigate the role of sirtuins in neointimal hyperplasia and VSMC function, the expression levels of SIRT1, SIRT3, SIRT5 and SIRT6 in carotid arteries of C57BL/6J mice were determined at 28 days after ligation. The results showed that the protein and mRNA levels of SIRT1, SIRT3, SIRT5 and SIRT6 in the ligated carotid arteries were markedly decreased compared with that of the control arteries (Figure 7A‐C). Then, we found that treatment of VSMCs with different concentrations of TGF‐β1 decreased the protein levels of SIRT1, SIRT3, SIRT5 and SIRT6 (Figure 7D,E). These findings suggest that SIRT1, SIRT3, SIRT5 and SIRT6 may be involved in the proliferation and migration of VSMCs.

**Figure 8:**
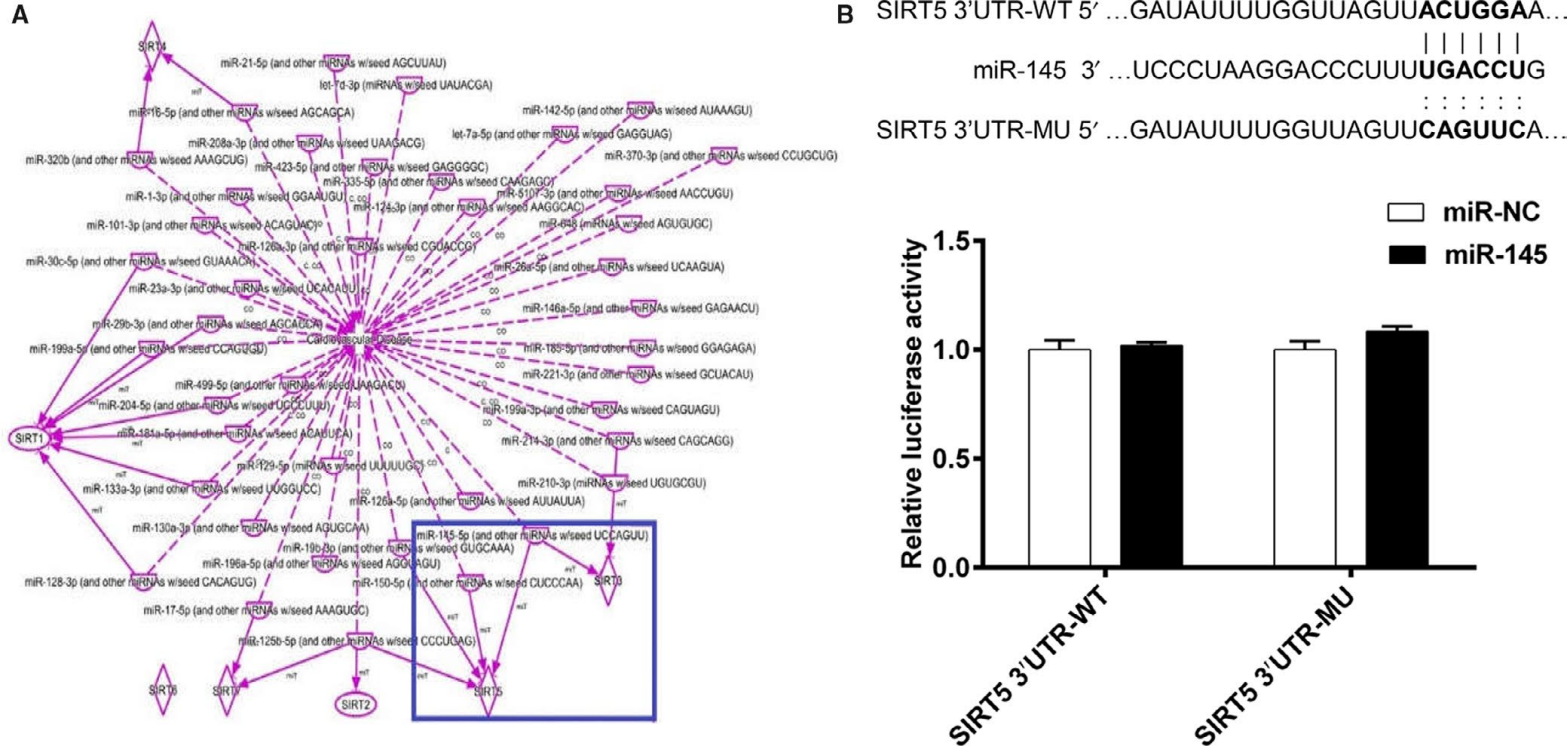
The results of the luciferase reporter assay. A, The relationships between microRNAs and sirtuins in cardiovascular diseases were examined by ingenuity pathway analysis. B, The luciferase reporter assay was performed to validate the direct targets of miR‐145

To clarify the regulatory effect of miR‐145 on sirtuins, the VSMCs with miR‐145 overexpression were stimulated with TGF‐β1 (5 ng/mL). The results showed that miR‐145 overexpression increased the expression levels of SIRT1, SIRT3, SIRT5 and SIRT6 in VSMCs, compared with the TGF‐β1 group (Figure 7F and G). Given the above results, we then proposed the hypothesis that miR‐145 may regulate the proliferation and migration of VSMCs through sirtuins. First, ingenuity pathway analysis was performed to analyse the relationships between microRNAs and sirtuins in cardiovascular diseases. The results showed that SIRT3 and SIRT5 may be the targets of miR‐145 (Figure 8A). Then, the luciferase reporter assay was performed to further validate whether miR‐145 interacts with SIRT5 directly. We found that there was no significant difference in the relative luciferase activity between cells cotransfected with miR‐145 and with the control oligonucleotide (Figure 8B). These results show that sirtuins are not the direct targets of miR‐145.

## 4 DISCUSSION

miR‐145 is considered as a potential biomarker and prognostic marker for progressing stages of cardiovascular diseases.^21^, ^22^ miR‐145 is the most abundant miRNA in VSMCs, and it controls vascular neointimal lesion formation.^3^, ^4^, ^5^ In the current study, we first observed that miR‐145 expression was decreased in the ligated carotid arteries compared with the control carotid arteries of mice. A previous study showed that miR‐145 expression was down‐regulated in the balloon‐injured rat carotid arteries.^4^ In addition, miR‐145 overexpression significantly reduced the neointimal thickness in a rabbit model of vein graft disease.^23^ We observed that TGF‐β1 decreased miR‐145 expression and promoted the proliferation and migration of VSMCs. It has been reported that miR‐145 expression was decreased in PDGF‐induced VSMC proliferation, and miR‐145 overexpression markedly inhibited VSMC proliferation.^3^ In the present study, we also found that miR‐145 overexpression inhibited the proliferation and migration of VSMCs, while the miR‐145 inhibitor caused the opposite effects on the proliferation and migration of VSMCs. These results suggest that miR‐145 regulates the proliferation and migration of VSMCs in intimal hyperplasia.

Autophagy is an evolutionarily conserved mechanism and linked to several cellular pathways, impacts the survival and function of VSMCs.^7^ Several studies have showed that the activation of autophagy contributes to the proliferation and migration of VSMCs.^8^, ^9^ The conversion of LC3, Beclin1 and p62 expression have been widely used to indicate the changes of autophagy.^24^ We first found that autophagy was activated by increasing the conversion of LC3 I to LC3 II and Beclin1 expression as well as decreasing p62 expression in the ligated carotid arteries of mice. Li *et al* also found that the conversion of LC3 I to LC3 II was significantly increased in neointimal lesions of mouse carotid arteries,^8^ which was consistent with our results. Then, our study indicated that TGF‐β1 promoted VSMC autophagy by increasing the autophagic flux and the conversion and expression of LC3, Beclin1 and p62, and autophagosome formation. It has been reported that miR‐145 regulated the autophagy of cardiomyocytes and then improved cardiac function and remodelling.^25^ Therefore, we speculate that miR‐145 may regulate the proliferation and migration of VSMCs via autophagy. Further study found that miR‐145 overexpression inhibited cell autophagy, whereas miR‐145 inhibition promoted autophagy in VSMCs stimulated with TGF‐β1. More importantly, autophagy inhibition augmented the inhibitory effects of miR‐145 on the proliferation and migration of VSMCs. Wu *et al* found that overexpression of miR‐145 significantly attenuated the proliferation and induced the autophagy and apoptosis of osteosarcoma cells.^26^ Another study showed that curcumin sensitized prostate cancer cells to radiation partly via miR‐143‐mediated autophagy inhibition.^27^ These studies provide further evidence that miR‐145 inhibits the proliferation and migration of VSMCs through autophagy.

It is generally accepted that miR‐145 exerts biological functions via its multiple target genes, including KLF5, TGFBR2 and CD40.^3^, ^10^, ^11^ Emerging evidence indicates that sirtuins are also the targets of miRNAs in cardiovascular diseases.^13^, ^14^, ^15^ In this study, we found that the expression of SIRT1, SIRT3, SIRT5 and SIRT6 was down‐regulated in the ligated carotid arteries of mice and VSMCs stimulated with TGF‐β1. Additionally, our study showed that miR‐145 overexpression increased the expression of SIRT1, SIRT3, SIRT5 and SIRT6 in VSMCs. It has been reported that SIRT1 plays a pivotal role in the regulation of cellular proliferation and invasion in atherosclerosis and angiogenesis.^28^, ^29^ The studies showed that SIRT1 was also the target of miR‐34a and miR‐138 in the proliferation, migration and differentiation of VSMCs.^13^, ^30^ Therefore, miR‐145 may regulate autophagy through sirtuins in the proliferation and migration of VSMCs. However, further studies found that sirtuins are not the direct targets of miR‐145. It has been reported that miR‐216a controls autophagy of vascular endothelial cells, but the autophagy associated gene 5 (ATG5) is not a direct target of miR‐216a.^31^ Le *et al* found that p53 is not a direct target of miR‐125b in mice; however, miR‐125b can indirectly affect p53 expression by upstream regulators.^32^ Therefore, we suppose that miR‐145 regulates the proliferation and migration of VSMCs through sirtuins. However, sirtuins are not the direct targets of miR‐145 in the proliferation and migration of VSMCs.

In conclusion, miR‐145 inhibits the proliferation and migration of VSMCs by suppressing the activation of autophagy. Sirtuins are not the direct targets of miR‐145 in the proliferation and migration of VSMCs. This study not only provides a theoretic and experimental basis for the regulatory effect of miR‐145 on autophagy of VSMCs in cardiovascular diseases, but also lays the foundation for its clinical application as a novel target for the prevention of cardiovascular disease. To further clarify the effects of miR‐145 on cellular proliferation, migration and autophagy in intimal hyperplasia of C57BL/6J mice. The mice are treated with Ad‐miR‐145, and then, the intimal hyperplasia and related cellular functions are determined in the future study. Further studies are also ongoing to validate the direct targets of miR‐145 in the proliferation and migration of VSMCs.

## CONFLICTS OF INTEREST

The authors confirm that there are no conflicts of interest.

## AUTHOR CONTRIBUTIONS

WRW, LFC and CXS performed the experiments and summarized the results. ZJ, FY and RW assisted in performing the experiments. LB and SHZ assisted in interpreting the data. WRW wrote the manuscript. EQL provided the supervision and assisted in writing the manuscript. All authors read and approved the final manuscript.

## Data Availability

The data that support the findings of this study are available from the corresponding author upon reasonable request.
